# Systemic nickel allergy syndrome

**DOI:** 10.1186/1939-4551-8-S1-A89

**Published:** 2015-04-08

**Authors:** Luciana Da Mata Perez, Alfeu Tavares França, José Roberto Zimmerman

**Affiliations:** 1 Alergoar,Luciana Da Mata Perez, Federal University of Rio De Janeiro, Brazil; 2 Hospital Universitário Clementino Fraga Filho Hucff-Ufrj, Brazil

## Background

Systemic nickel allergy syndrome (SNAS) is characterized by contatc dermatitis associated with systemic symptoms after ingestion of foods containing nickel.

## Methods

Demonstrate SNAS which is characterized by contact dermatitis to nickel and systemic reactions after ingestion of rich foods nickel.We evaluated adult patients with ages between 18 and 65 years, positive patch test for nickel, grades 3 and 4, and who had symptoms suggestive of SNAS.These patients had eczematous lesions of contact dermatitis did not disappear. That even with the exclusion of products containing nickel in this composition worsened without new skin exposure to these products. The study was made with 331 patients selected among January of 2012 to April 2014, and 87 of them related worsening with ingestion of food that they didn't know who to identify.

## Results

During the elimination diet of rich food in nickel for 60 days, patients reported significant improvement and, or abscense of symptoms, mainlt gastrointestinal and cutaneous. After the period, the diet was reintroduced in 43 pacients, and others 44, maintained the exclusion diet for over 60 days. At the first group, there was a reset of symptoms between the seventh and the twentieth day free diet. The second group maintained the exclusion diet, continued without symptoms.

## Conclusions

The SNAS is fully associated based foods rich in nickel, found in cereals (oats, barley, corn, soy, whole wheat flour, beans), fruits (apricot, cherry, grape, pear, fig,melon, banana, plum, kiwi), vegetables (broccoli, onion, spinach, lettuce, chicory, asparagus, cauliflower), meats (cooked ham), fish and seafood (salmon, hake, octopus, oysters, lobster,calamari), sweet (cocoa and derivatives, brioches and clad masses) and also related to household utensils used in food preparation. Unfortunately, we still lack the oral vaccine for nickel, avaiable only in Europe, the results are good. Therefore, we can only control our patients with an exclusion diet. Patients with contact dermatitis to nickel, gastrointestinal symptoms and urticaria, must be investigated about the ingestion of foods containing nickel.

